# Influenza-Associated Hospitalizations During a High Severity Season — Influenza Hospitalization Surveillance Network, United States, 2024–25 Influenza Season

**DOI:** 10.15585/mmwr.mm7434a1

**Published:** 2025-09-11

**Authors:** Alissa O’Halloran, Jennifer Whitmill Habeck, Matthew Gilmer, Ryan Threlkel, Shua J. Chai, Brenna Hall, Isaac Armistead, Nisha B. Alden, James Meek, Kimberly Yousey-Hindes, Kyle P. Openo, Lucy S. Witt, Maya L. Monroe, Patricia A. Ryan, Lauren Leegwater, Sue Kim, Melissa McMahon, Ruth Lynfield, Khalil Harbi, Murtada Khalifa, Caroline McCahon, Grant Barney, Bridget J. Anderson, Christina B. Felsen, Brenda L. Tesini, Nancy E. Moran, Denise Ingabire-Smith, Melissa Sutton, M. Andraya Hendrick, William Schaffner, H. Keipp Talbot, Andrea George, Hafsa Zahid, Shikha Garg, Catherine H. Bozio

**Affiliations:** ^1^Influenza Division, National Center for Immunization and Respiratory Diseases, CDC; ^2^Goldbelt Professional Services, Chesapeake, Virginia; ^3^California Emerging Infections Program, Oakland, California; ^4^Division of State and Local Readiness, Office of Readiness and Response, CDC; ^5^Colorado Department of Public Health and Environment; ^6^Connecticut Emerging Infections Program, Yale School of Public Health, New Haven, Connecticut; ^7^Division of Infectious Diseases, Emory University School of Medicine, Atlanta, Georgia; ^8^Georgia Emerging Infections Program, Atlanta, Georgia; ^9^Maryland Department of Health, Baltimore, Maryland; ^10^Michigan Department of Health and Human Services; ^11^Minnesota Department of Health; ^12^North Carolina Division of Public Health; ^13^New Mexico Department of Health; ^14^New York State Department of Health; ^15^University of Rochester School of Medicine and Dentistry, Rochester, New York; ^16^Ohio Department of Health; ^17^Public Health Division, Oregon Health Authority, Portland, Oregon; ^18^Vanderbilt University Medical Center, Nashville, Tennessee; ^19^Salt Lake County Health Department.

**Summary**

**What is already known about this topic?**

Seasonal influenza causes substantial annual U.S. morbidity and mortality.

**What is added by this report?**

Among a surveillance sample of the U.S. population, 2024–25 was a high severity influenza season. The cumulative influenza-associated hospitalization rate was the highest since 2010–11. During the 2024–25 season, the percentages of patients admitted to an intensive care unit (16.8%) and who received invasive mechanical ventilation (6.1%) were similar to past seasons’ estimates. Approximately one third of hospitalized patients were vaccinated. Children aged 5–17 years were the lowest percentage of hospitalized patients receiving antiviral treatment (61.6%).

**What are the implications for public health practice?**

All persons aged ≥6 months should receive an annual seasonal influenza vaccine. All hospitalized patients with suspected or confirmed influenza should receive timely antiviral treatment to reduce the risk for influenza-associated complications.

## Abstract

The U.S. 2024–25 influenza season was a high-severity season characterized by co-circulation of influenza A(H1N1)pdm09 and A(H3N2) viruses. Data from the Influenza Hospitalization Surveillance Network covering 9% of the U.S. population, were analyzed to compare laboratory-confirmed influenza-associated hospitalization rates and patient clinical characteristics from the 2024–25 season with data from past seasons. Based on preliminary data from influenza-associated hospital admissions from October 1, 2024, through April 30, 2025, the cumulative influenza-associated hospitalization rate (127.1 influenza-associated hospitalizations per 100,000 population) had surpassed all end-of-season rates during the period beginning with the 2010–11 season. Cumulative 2024–25 season rates were highest among persons aged ≥75 years (598.8). Across age groups, hospitalization rates during the 2024–25 season were 1.8 to 2.8 times higher than median historical rates during the period beginning with the 2010–11 season. Among hospitalized patients, 32.4% had received an influenza vaccine, and 84.8% received antiviral treatment, though children and adolescents aged 5–17 years had the lowest proportion of antiviral receipt (61.6%). Similar to past seasons, most patients hospitalized with influenza during the 2024–25 season (89.1%) had one or more underlying medical conditions, 16.8% were admitted to an intensive care unit, 6.1% received invasive mechanical ventilation, and 3.0% died in hospital. Seasonal influenza viruses can cause severe disease, particularly among persons who are at higher risk for complications. CDC recommends that all persons aged ≥6 months who do not have contraindications receive an annual influenza vaccine and that all hospitalized patients with influenza receive timely antiviral treatment to reduce the risk for complications.

## Introduction

CDC classified the U.S.2024–25influenzaseason as a high-severity season for all age groups, making it the first such season since 2017–18. CDC assesses seasonal severity annually by comparing the current season’s influenza activity with thresholds based on peak influenza activity in past seasons for three surveillance indicators, including laboratory-confirmed influenza-associated hospitalization rates from the Influenza Hospitalization Surveillance Network (FluSurv-NET) (*1*). FluSurv-NET data were used to describe influenza-associated hospitalization rates, clinical characteristics, and outcomes in all age groups, comparing the 2024–25 influenza season with past seasons.

## Methods

### Data Source

FluSurv-NET conducts population-based surveillance for laboratory-confirmed influenza-associated hospitalizations in patients of all ages in approximately 300 acute care hospitals in 14 states,* covering 9% of the U.S. population (*2*). A FluSurv-NET patient was defined as a person who 1) was a resident of the surveillance catchment area, 2) had a hospital admission during October 1–April 30^†^ of a given season, and 3) received a positive influenza test result ≤14 days before or anytime during hospitalization. Preliminary 2024–25 season’s data were updated on September 2, 2025; data were 99% complete for rate estimation, 99% complete for clinical data (excluding influenza vaccination), and 85% complete for review of vaccination data.

### Influenza-Associated Hospitalization Rate Estimation

Cumulative influenza-associated hospitalization rates (hospitalizations per 100,000 population)^§^ were stratified by season (2010–11 through 2024–25, excluding 2020–21), age group (0–4, 5–17, 18–49, 50–64, 65–74, and ≥75 years), influenza virus type (A or B) and influenza A virus subtype (H1N1pdm09 or H3N2). The 2020–21 season is excluded from this report because there were not enough influenza-associated hospitalizations reported to estimate age-stratified rates or clinical estimates; the 2020–21 cumulative hospitalization rate among persons of all ages is available from CDC’s FluView. Median end-of-season cumulative rates across the 2010–11 through 2023–24 seasons captured rate variability across seasons, and a rate ratio was estimated by dividing the 2024–25 rate by the historical median rate. Peak weekly influenza-associated hospitalization rates were stratified by season.

### Imputation of Missing Influenza A Subtype

Influenza A virus subtype was missing for a median 56% (IQR 48%–64%, range = 38%–72%) of patients across seasons. Influenza A subtype data could have been missing at random or not at random; nevertheless, multiple imputation procedures were performed over 70 full datasets via logistic regression, with site, age group, and admission month as predictors (*3*). In the 2021–22 season, >99% of influenza A cases in patients in FluSurv-NET were A(H3N2); as such, in the 2021–22 season, missing influenza A subtype for FluSurv-NET cases was assumed to be A(H3N2). Imputed subtype rates and 95% CI were pooled using SAS Proc MIAnalyze.

### Clinical Data

Clinical data were abstracted from medical records using a case report form (CRF) for sampled FluSurv-NET cases. During the 2017–18 through 2024–25 seasons,^¶^ patients were stratified by site, age, admission month, and outcome (died in hospital versus discharged alive), and random samples were drawn within each stratum for CRF completion (*2*). Influenza vaccination status was obtained from up to four sources: patient medical chart, state immunization registry, patient’s primary care provider, and patient or proxy interview. Preliminary 2024–25 clinical data were further limited to discharged or deceased sampled patients with a completed CRF.

### Weighting and Analysis of Clinical Characteristics of Hospitalized Patients

Sample weights were calculated as the inverse probability of selection based on the percentage of the sample drawn from all hospitalized cases. Weighted proportions of 2024–25 patient clinical characteristics, stratified by age group, were compared with the range of weighted proportions across the 2017–18 through 2023–24 seasons. Data were analyzed using SAS 9.4 (SAS Institute).

FluSurv-NET sites obtained human subjects and ethics approval from their respective state health department, academic partners, and participating hospital institutional review boards as needed. This activity was reviewed by CDC, deemed not research, and was conducted consistent with applicable federal law and CDC policy.**

## Results

### Influenza-Associated Hospitalizations During the 2024–25 Season

**FIGURE 1:**
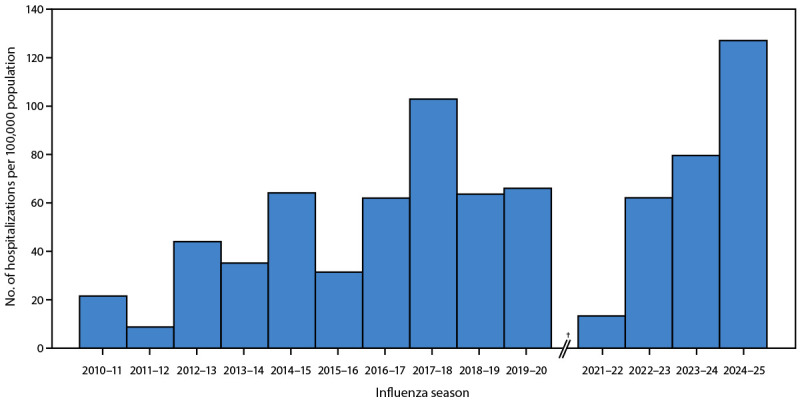
Cumulative laboratory-confirmed influenza-associated hospitalization rates,* by influenza season — Influenza Hospitalization Surveillance Network, United States, 2010–11 through 2024–25 seasons^†^ * End-of-season rate. ^†^ Data from the 2020–21 season were excluded from the analysis because of low numbers. The median hospitalization rate (based on end-of-season cumulative rates from 2010–11 through 2023–24) was 62.0 (IQR = 31.4–64.1) hospitalizations per 100,000 population.

From October 1, 2024, through April 30, 2025, FluSurv-NET identified 38,960 influenza-associated hospitalizations. The overall 2024–25 cumulative hospitalization rate (127.1) surpassed end-of-season rates from past seasons (median 2010–11 through 2023–24 = 62.0, range = 8.7 [2011–12] to 102.9 [2017–18]) (Figure 1). The weekly influenza hospitalization rate peaked at 13.5 per 100,000 in early February, representing the highest weekly rate observed during the period since the 2010–11 season (range = 1.1 [2011–12] to 10.2 [2017–18]) (SupplementaryFigure).

Compared with median historical cumulative rates, rates during the 2024–25 season were 1.8 to 2.8 times higher across all age groups (SupplementaryTable1). The 2024–25 influenza-associated hospitalization rate was highest in patients aged ≥75 years (598.8) and lowest in those aged 5–17 years (39.3). Hospitalization rates in patients aged <75 years were higher during the 2024–25 season compared with rates during past seasons; among patients aged ≥75 years, the 2024–25 rate (598.8) was the second highest rate after the 2017–18 season (726.5).

### Hospitalization Rates Among Patients with Influenza A(H1N1)pdm09, Influenza A(H3N2), and Influenza B

**FIGURE 2:**
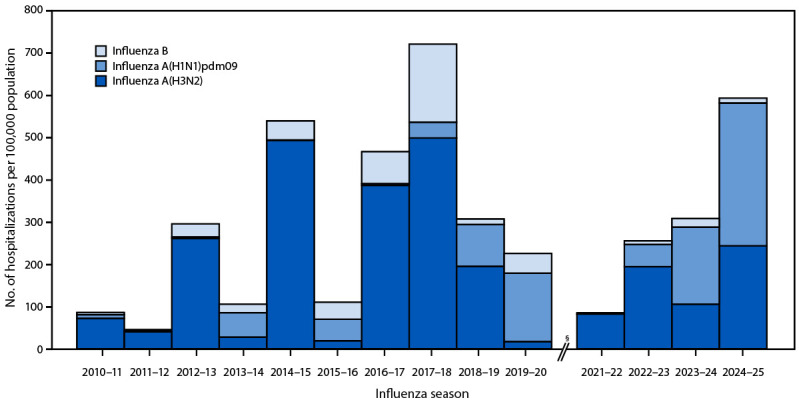
Cumulative influenza-associated hospitalization rates* among adults aged ≥75 years, by influenza type and subtype^†^ and influenza season — Influenza Hospitalization Surveillance Network, United States, 2010–11 through 2024–25 seasons^§^ * End-of-season rate. ^†^ Influenza A subtype information could be missing at random or not at random. Data were imputed using multiple imputation via a logistic regression model in which age, site, and month of admission were predictors. ^§^ Data from the 2020–21 season were excluded from the analysis because of low numbers.

Cumulative 2024–25 hospitalization rates (95% CIs) were estimated to be higher among patients with influenza A virus infections (122.0 [120.7–123.2]) than among those with influenza B (4.8 [4.5–5.0]); hospitalization rates were also higher among those with A(H1N1)pdm09 (72.2 [71.0–73.5]) than those with A(H3N2) (49.5 [48.4–50.6]) (SupplementaryTable2). Subtype-specific hospitalization rates differed by age group. Among patients aged ≥75 years, the estimated 2024–25 A(H1N1)pdm09 hospitalization rate (339.5 [329.3–349.7]) was higher than that for A(H3N2) (244.8 [235.6–254.0]). During 2017–18, the estimated A(H3N2) hospitalization rate was higher (501.3 [489.7–512.8]) than the rate for A(H1N1)pdm09 (37.6 [32.8–42.3]) in this age group (Figure 2).

### Underlying Medical Conditions, Treatments, and Outcomes Among Hospitalized Influenza Patients

Among 10,269 randomly sampled patients hospitalized with influenza who had the CRF completed in the 2024–25 season, the median number of underlying medical conditions ranged from zero to three per patient across age groups, similar to historical median numbers. The most common underlying condition was asthma among children aged 0–4 years and 5–17 years (14.0% and 35.9%, respectively); obesity among adults aged 18–49 years (43.9%); chronic metabolic disease among adults aged 50–64 years (45.6%), including diabetes mellitus, adrenal disorders, glycogen or other storage diseases, hyperfunction or hypofunction of the pituitary gland, inborn errors of metabolism, metabolic syndrome, parathyroid dysfunction, and thyroid dysfunction; and cardiovascular disease among adults aged 65–74 and ≥75 years (57.0% and 69.3%, respectively) (Table). Among patients during the 2024–25 season, 16.8% were admitted to an intensive care unit (ICU), 6.1% received invasive mechanical ventilation, and 3.0% died in hospital, similar to prevalences during seasons since 2017–18 (range of patients with ICU admission, mechanical ventilation, and death = 14.3%–18.2%, 4.9%–6.5%, and 2.3%–2.9%, respectively). As in previous seasons, the most frequent complications during hospitalization during the 2024–25 season were pneumonia (30.0%), sepsis (18.5%), and acute renal failure (18.1%). During the 2024–25 season, 32.4% of patients had received an influenza vaccine, similar to past seasons (range = 29.3%–48.2%), with 28.5% of patients missing preliminary information on influenza vaccination status in 2024–25, higher than the range of missing vaccination in past seasons (10.4%–22.3%). In addition, 84.8% of patients received influenza antiviral treatment; the range during previous seasons was 76.4%–92.4%. Children aged 5–17 years and adults aged ≥75 years accounted for the lowest (61.6%) and highest (88.3%) percentages of patients who received antiviral treatment, respectively. In previous seasons, the ranges of percentage of antiviral treatment in these age groups were 56.5%–84.5% and 84.0%–94.2%, respectively.

**TABLE:** Demographic and clinical characteristics of laboratory-confirmed influenza-associated hospitalizations, overall and by age group among sampled* patients — Influenza Hospitalization Surveillance Network, United States, 2017–18 through 2024–25 seasons

Characteristic	Total		Age group, yrs											
			0–4		5–17		18–49		50–64		65–74		≥75	
	2017–18 to 2023–24^†^	2024–25^§^ N = 10,269	2017–18 to 2023–24^†^	2024–25^§^ n = 1,566	2017–18 to 2023–24^†^	2024–25^§^ n = 1,270	2017–18 to 2023–24^†^	2024–25^§^ n = 1,166	2017–18 to 2023–24^†^	2024–25^§^ n = 1,758	2017–18 to 2023–24^†^	2024–25^§^ n = 1,655	2017–18 to 2023–24^†^	2024–25^§^ n = 2,854
	Range of weighted proportions	Weighted proportion	Range of weighted proportions	Weighted proportion	Range of weighted proportions	Weighted proportion	Range of weighted proportions	Weighted proportion	Range of weighted proportions	Weighted proportion	Range of weighted proportions	Weighted proportion	Range of weighted proportions	Weighted proportion
**Sex**														
Female	**51.8–54.9**	**52.7**	41.8–45.1	45.5	41.5–46.2	41.6	57.4–64.4	54.9	48.6–54.4	51.5	48.4–52.1	48.5	53.2–58.4	57.9
Male	**45.1–48.2**	**47.3**	54.9–58.2	54.5	53.8–58.5	58.4	35.6–42.6	45.1	45.6–51.4	48.5	47.9–51.6	51.5	41.6–46.8	42.1
**Race and ethnicity**														
A/PI, non-Hispanic	**3.8–5.2**	**5.2**	4.5–7.0	7.5	2.7–5.6	3.9	4.1–4.8	5.6	2.8–3.9	4.0	3.3–5.6	4.3	3.8–7.3	6.3
AI/AN, non-Hispanic	**0.5–1.2**	**0.9**	0.7–1.9	1.9	1.0–1.3	1.1	0.7–1.8	1.7	0.2–1.5	1.7	0.3–1.7	0.1	0.1–0.8	0.2
Black or African American, non-Hispanic	**19.4–26.8**	**24.6**	24.4–31.9	26.0	24.9–31.9	28.0	28.6–38.9	37.6	26.3–31.0	26.5	14.9–23.3	26.6	7.7–13.0	13.9
White, non-Hispanic	**51.9–61.9**	**54.1**	25.4–31.4	31.9	34.7–39.4	39.1	33.5–44.6	34.7	48.6–56.9	51.0	59.6–69.0	60.3	70.4–74.1	69.0
Multiple races, non-Hispanic	**0.3–0.7**	**1.0**	0.4–1.8	1.7	0.7–2.0	2.5	0.4–0.8	0.7	0.2–1.2	1.1	0.1–0.5	0.5	0.1–0.5	1.0
Hispanic or Latino	**7.5–16.4**	**11.2**	23.9–29.9	27.6	20.1–23.7	21.8	12.6–22.9	14.9	7.3–12.3	13.7	4.9–13.0	6.3	4.3–10.7	6.5
Unknown race	**2.8–5.2**	**3.0**	5.0–8.2	3.5	3.5–5.7	3.5	3.3–6.4	4.9	1.9–5.3	2.1	2.2–5.4	2.0	2.8–4.9	3.1
**Respiratory signs or symptoms at admission**	**84.7–92.9**	**86.6**	88.8–93.4	91.6	83.1–90.2	88.3	76.3–91.7	82.5	87.6–94.1	89.2	86.2–93.5	89.2	85.1–92.7	84.5
Underlying medical condition^¶^	**85.7–90.8**	**89.1**	37.6–44.1	39.1	68.5–73.5	70.4	84.2–88.4	85.0	91.2–94.9	92.6	93.2–95.9	94.8	94.0–96.9	95.9
Median no. of underlying medical conditions**	**2–2**	**2 (1–3)**	0–0	0 (0–1)	1–1	1 (0–2)	1–2	2 (1–3)	2–3	3 (1–4)	3–3	3 (1–4)	2–3	3 (2–4)
**No. of underlying medical condition categories**														
0	**10.1–20.4**	**11.7**	60.0–66.9	64.6	27.7–34.1	30.7	12.7–19.6	16.1	6.4–16.0	7.8	5.1–12.7	6.0	4.2–8.7	4.6
1	**20.8–23.3**	**20.6**	22.4–27.3	24.4	36.3–40.0	40.8	29.6–34.1	28.1	16.0–20.6	19.9	13.3–16.7	19.3	13.5–17.9	13.8
2	**21.6–24.9**	**22.0**	6.5–7.8	7.0	16.4–22.6	18.2	24.8–28.8	27.2	22.3–26.1	20.7	19.2–25.0	19.2	24.0–26.7	24.7
3	**16.1–21.8**	**21.0**	1.1–3.3	3.0	5.4–8.8	6.1	13.2–15.8	17.3	17.4–23.6	23.1	20.3–25.9	21.8	23.1–26.1	26.2
≥4	**19.5–22.8**	**24.7**	0.4–2.2	1.0	2.5–4.2	4.2	9.1–11.8	11.4	24.2–34.3	28.5	27.9–34.5	33.7	23.9–29.8	30.7
**Underlying medical conditions**														
Asthma	**19.8–23.3**	**21.6**	12.0–15.9	14.0	32.2–40.5	35.9	26.4–34.0	31.3	22.8–30.0	25.9	16.9–19.1	18.3	12.6–14.9	14.5
Chronic lung disease	**27.6–32.2**	**32.5**	2.5–6.2	4.8	6.4–8.5	7.8	10.1–13.5	13.0	35.3–46.4	40.4	36.9–48.9	50.1	28.5–38.5	34.3
Chronic metabolic disease^††^	**35.0–41.5**	**40.2**	1.7–4.4	2.1	5.7–7.8	4.3	19.9–25.8	27.3	42.0–48.7	45.6	46.7–51.5	46.6	47.6–50.2	51.1
Diabetes	**25.5–30.3**	**30.7**	0.1–0.8	0.3	2.2–5.5	1.6	15.0–19.8	21.2	33.8–40.0	39.1	37.8–41.0	38.5	31.2–35.8	34.5
Cardiovascular disease	**38.2–47.8**	**45.9**	6.0–8.7	8.7	3.9–7.7	7.2	11.7–17.0	17.8	39.1–44.8	40.6	53.1–59.6	57.0	66.4–73.3	69.3
Blood disorder	**2.2–5.2**	**5.0**	2.7–6.0	3.5	6.5–9.0	8.2	3.0–5.5	3.9	1.6–6.1	5.9	1.7–5.7	5.0	1.4–5.0	4.7
Immunocompromising condition	**10.4–17.6**	**13.8**	3.0–5.6	2.5	6.3–11.3	7.0	6.8–16.1	10.9	14.4–22.4	18.4	14.6–22.4	16.1	9.9–16.1	13.7
Liver disease	**4.6–6.3**	**6.1**	0.4–0.9	0.6	0.9–2.0	2.2	4.8–7.1	5.0	8.4–9.8	9.4	6.0–9.1	9.3	1.4–5.2	4.0
Neurologic or neuromuscular disorder	**19.8–22.8**	**23.6**	11.2–15.1	11.2	16.9–23.4	23.6	11.1–14.0	17.1	13.8–18.3	17.9	18.3–21.3	20.6	31.3–34.0	34.6
Obesity	**37.1–40.8**	**35.1**	13.1–16.4	9.5	17.8–22.6	15.1	45.7–52.7	43.9	48.2–55.0	43.4	41.5–45.6	38.6	26.0–29.3	27.5
Renal disease	**15.2–20.0**	**20.6**	0.4–1.5	0.6	1.2–3.9	2.6	5.7–9.9	10.1	14.5–17.8	18.2	21.3–27.8	21.6	26.0–29.3	33.1
Pregnant**^§§^**	**20.0–37.6**	**22.8**	NA	NA	7.5–16.0	23.1	20.3–38.7	22.8	NA	NA	NA	NA	NA	NA
**Received seasonal influenza vaccine^¶¶^**														
Yes	**29.3–48.2**	**32.4**	18.1–41.8	26.7	18.3–40.6	25.8	14.2–29.9	11.3	24.2–39.7	25.5	36.7–55.9	33.4	43.6–64.1	50.3
No	**34.3–55.5**	**39.0**	40.2–67.6	56.3	47.0–67.4	55.2	50.1–67.9	52.9	42.4–58.3	43.3	31.4–50.2	36.7	23.3–41.9	25.0
Unknown	**10.4–22.3**	**28.5**	5.4–19.2	17.0	6.2–21.0	19.0	13.8–29.4	35.8	11.6–24.6	31.2	9.2–22.3	30.0	9.7–20.0	24.8
**Treatments and outcomes**														
Received antiviral treatment	**76.4–92.4**	**84.8**	60.2–86.8	65.4	56.5–84.5	61.6	73.1–92.1	84.9	79.7–92.0	87.0	81.0–92.6	87.6	84.0–94.2	88.3
Admitted to ICU	**14.3–18.2**	**16.8**	17.5–22.2	18.0	18.2–24.9	24.0	11.8–17.9	18.7	16.7–20.6	17.9	12.9–19.8	17.2	10.8–14.7	13.3
ECMO	**0.1–0.5**	**0.4**	0.1–0.6	0.7	0.1–1.1	0.4	0.5–0.8	0.7	0.1–1.3	0.7	0.1–0.2	0.3	0–0.1	0.1
IMV	**4.9–6.5**	**6.1**	3.7–5.7	4.6	3.1–5.6	5.2	3.8–7.2	6.6	6.3–9.3	7.5	5.1–7.3	7.3	3.2–4.2	4.3
Length of stay, days**	**3–3**	**3 (2**–**6)**	2–2	2 (1–3)	2–2	2 (1–4)	2–3	3 (2–5)	3–3	3 (2–7)	3–4	4 (2–7)	4–4	4 (2–7)
Died in hospital	**2.3–2.9**	**3.0**	0.2–0.6	0.6	0.4–1.1	0.6	0.1–1.6	1.5	2.4–3.4	3.1	2.6–3.9	3.3	4.2–4.8	4.5
Complications***														
Pneumonia	**21.9–28.2**	**30.0**	8.4–24.2	21.9	10.7–20.6	22.9	14.2–27.5	28.8	27.1–31.7	34.8	26.1–29.9	27.2	28.7–29.9	31.7
Sepsis	**11.6–19.0**	**18.5**	1.8–2.6	2.8	2.9–4.7	6.0	12.8–22.9	21.8	12.0–24.2	22.9	15.2–20.8	18.7	13.0–20.2	17.9
Acute renal failure/acute kidney injury	**12.8–16.2**	**18.1**	0.4–1.5	1.5	3.3–4.9	5.7	6.4–11.5	11.1	15.6–19.2	19.5	17.5–20.3	26.2	17.3–21.8	20.4
Chronic obstructive pulmonary disease exacerbation	**10.9–14.9**	**13.2**	0.1–0.1	0	0–0	0	1.5–3.2	1.9	15.6–22.4	17.0	20.4–24.3	24.1	13.0–16.8	14.1
Asthma exacerbation	**6.0–8.8**	**7.3**	4.3–6.7	7.9	11.5–19.7	16.6	11.3–16.5	12.4	6.8–10.8	8.6	3.2–5.7	3.8	2.8–3.5	4.1
Congestive heart failure	**4.5–5.8**	**6.4**	0.1–0.3	0.3	0.1–0.3	0	1.5–2.7	2.7	4.5–6.8	5.6	5.0–7.8	9.1	7.1–10.0	9.3
Acute myocardial infarction	**1.5–2.1**	**2.7**	0–0	0	0.1–0.1	0	0.5–0.9	1.5	1.3–2.6	2.5	2.1–3.5	4.8	2.5–3.6	3.1
Bacteremia	**1.5–2.3**	**2.4**	0.3–0.9	0.5	0.8–2.0	0.6	1.4–2.6	4.3	1.7–3.5	2.5	1.7–2.9	1.1	1.0–2.2	2.5
Diabetic ketoacidosis	**0.7–1.6**	**1.8**	0.1–0.4	0.3	1.2–3.6	0.9	3.1–3.8	5.0	0.6–2.5	2.3	0.3–1.1	1.4	0.1–0.3	0.3
Rhabdomyolysis	**1.0–1.7**	**1.7**	0.1–0.8	0.7	2.2–5.6	4.2	0.4–2.0	1.0	0.3–1.1	1.1	0.8–1.6	1.9	0.5–1.9	2.1
Seizure	**1.0–2.9**	**1.5**	3.7–7.9	5.8	2.7–5.1	4.5	1.0–2.6	2.7	0.6–3.5	0.9	0.6–1.9	0.7	0.3–2.7	0.7
Acute respiratory distress syndrome	**0.5–1.7**	**1.4**	1.1–2.3	1.5	0.8–1.6	1.5	0.3–2.9	2.3	0.6–2.7	2.5	0.5–1.3	0.9	0.2–1.3	0.4
Bronchiolitis	**0.9–2.1**	**1.4**	11.1–21.4	17.5	0.3–0.8	0.3	0.1–0.9	0.5	0.2–0.6	1.2	0.1–0.5	0.6	0.1–0.3	0.2
Stroke	**0.4–0.9**	**1.0**	0.1–0.2	0.1	0.1–0.3	0.1	0.1–0.5	0.8	0.4–1.2	1.3	0.2–1.4	2.0	0.6–1.7	0.5
Acute myocarditis	**0.1–0.3**	**0.1**	0.1–0.1	0	0.1–0.7	0.5	0–0.3	0	0–0.3	0.1	0.1–0.5	0	0–0.1	0.1

**Abbreviations:** AI/AN = American Indian or Alaska Native; A/PI = Asian or Pacific Islander; ECMO = extracorporeal membrane oxygenation; ICU = intensive care unit; IMV = invasive mechanical ventilation; NA = not available.

* Patients were randomly sampled by site, age group, month of admission, and outcome for medical chart review.

^†^ The minimum and maximum proportions from 2017–18 through 2023–24 are included for comparison with the 2024–25 season. The 2020–21 season was excluded.

^§^ The 2024–25 denominator includes patients with a discharge or death date and a completed chart review.

^¶^ Underlying medical conditions include asthma, chronic lung disease, chronic metabolic disease, diabetes, cardiovascular disease, blood disorder, immunocompromising condition, liver disease, neurologic/neuromuscular disorder, obesity, renal disease, and pregnancy.

** The median and IQR are presented for the 2024–25 season. For the other seasons, the highest and lowest median are presented.

**^††^** Chronic metabolic disease included diabetes mellitus, adrenal disorders, glycogen or other storage diseases, hyperfunction or hypofunction of the pituitary gland, inborn errors of metabolism, metabolic syndrome, parathyroid dysfunction, and thyroid dysfunction.

^§§^ The denominator used is women aged 15–44 years (2017–18 through 2021–22) or women aged 15–49 years (2022–23 through 2024–25).

^¶¶^ Vaccination data were collected from persons aged ≥6 months. The denominator used for children aged 6 months–4 years was 1,316.

*** Complications are based on discharge diagnoses documented in the medical chart.

## Discussion

Among a 9% surveillance sample of the US population, the peak weekly influenza-associated hospitalization rate during the 2024–25 U.S. influenza season, surpassed all past peak weekly rates since 2010–11. This season’s peak weekly influenza-associated hospitalization rate also surpassed peak weekly COVID-19 hospitalization rates since January 2022 when the SARS-CoV-2 Omicron variant emerged and COVID-19–associated hospitalizations surged (RespiratoryVirusHospitalizationSurveillanceNetwork(RESP-NET)|CDC; COVID-19, influenza, and respiratory syncytial virus (RSV)-associated hospitalization rates are not adjusted for testing practices). Cumulative influenza hospitalization rates were the highest since 2010–11 in all age groups except those aged ≥75 years. In all age groups, 2024–25 rates were 1.8–2.8 times higher than median historical rates. The cumulative influenza-associated hospitalization rate among persons of all ages was also higher than that of COVID-19 or RSV this season.

High rates observed during the 2024–25 season could have been driven by recent lower influenza vaccination coverage in the general population (WeeklyFluVaccinationDashboard|CDC), as well as virus characteristics. The distribution of 2024–25 influenza virus A subtypes might partially explain why, in contrast to other age groups, rates among persons aged ≥75 years were not the highest compared with past seasons. Since persons aged ≥75 years retain immunologic protection against A(H1) viruses from early childhood exposures, they have historically experienced more severe illness and death in A(H3N2)-predominant seasons (*4*). In 2017–18, the last season classified as highly severe for all age groups, circulating influenza A viruses were predominantly A(H3N2) (84%), whereas in 2024–25, both A(H3N2) and A(H1N1) viruses co-circulated equally (FluViewWeeklyInfluenzaSurveillanceReport|CDC). Annual influenza vaccination for persons aged ≥6 months and early initiation of antiviral treatment for patients with influenza who are at higher risk for complications can help prevent adverse outcomes (*5*,*6*). Nonpharmacologic measures, such as hand washing, might also prevent transmission (*6*,*7*).

Among patients hospitalized with influenza, the number of underlying medical conditions and the most common conditions were similar to those from previous seasons, underscoring the increased risk for influenza-associated complications in persons with comorbidities (PeopleatIncreasedRiskforFluComplications|CDC). However, nearly 11% of all patients hospitalized with influenza did not have any underlying medical conditions, highlighting that healthy individuals may also experience influenza-associated hospitalizations or complications. Given similar prevalences of severe disease indicators this season compared with past seasons, the 2024–25season’sseverity was likely driven by higher incidence rather than atypical clinical severity. Higher estimated numbers of U.S. influenza-associated hospitalizations likely resulted in higher absolute numbers of hospitalized patients requiring ICU beds and ventilators this season compared with past seasons.

Influenza antiviral treatment is associated with improved patient outcomes including in-hospital survival (*6*,*8*) and is recommended for all patients hospitalized with influenza (InfluenzaAntiviralMedications:SummaryforClinicians|CDC) (*9*). Recent notable declines in antiviral treatment have been previously described in hospitalized patients with influenza (*2**,**10*). While further declines were not observed, influenza antiviral treatment rates remain suboptimal, particularly among children and adolescents, since all patients hospitalized with influenza should receive prompt antiviral treatment, provided they do not have contraindications.

### Limitations

The findings in this report are subject to at least five limitations. First, influenza-associated hospitalizations rates might be underestimated because of clinician-driven influenza testing. Second, influenza A subtype was missing for a median 56% (IQR 48%-64%; range 38%–72%) of patients, and the missingness could have been non-random. Thus the hospitalization rate estimates for A(H1N1)pdm09 and A(H3N2) subtypes derived from multiple imputation procedures using 3 predictor variables (site, age, month) are likely biased and should be interpreted cautiously. However this concern might be minimized because the imputed subtype distribution of hospitalizations by age in all seasons since 2015–16 was similar to the subtype distribution in U.S. Public Health Laboratory data, in which data missingness was much lower (1%–20% for 2015–16 through 2024–25 excluding the 2020–21 season; data by patient/person age unpublished, but overall Public Health Laboratory data are available at National,Regional,andState-LevelOutpatientIllnessandViralSurveillance|CDC). Third, nonclinical factors, such as hospital admission thresholds, that might have resulted in changes in the number of hospitalizations, could not be measured; however, the percentage of patients likely admitted for influenza-like illness has increased from 79% in 2021–22 to 87% in 2024–25. Fourth, because influenza vaccination history is subject to more reporting delays than other outcomes in the analysis, 28.5% of hospitalized patients were missing this season’s influenza vaccination status. Finally, the FluSurv-NET catchment area represents 9% of the U.S. population and might not be generalizable to the entire U.S. population; hospitalization rates in this report represent the FluSurv-NET catchment area.

### Implications for Public Health Practice

During the 2024–25 U.S. influenza season, the overall and peak weekly influenza-associated hospitalization rates were the highest recorded since the period beginning with the 2010–11 season. All persons aged ≥6 months who did not have contraindications are recommended to receive annual influenza vaccination (*5*,*7*). To reduce the risk of influenza-associated complications, early initiation of antiviral treatment is recommended for all hospitalized patients with suspected or confirmed influenza illness.

## Footnotes

* Data from the 2024–25 season included those from selected counties in 14 U.S. states (California, Colorado, Connecticut, Georgia, Maryland, Michigan, Minnesota, New Mexico, New York, North Carolina, Ohio, Oregon, Tennessee, and Utah). From the 2010–11 through 2023–24 influenza seasons, the number of sites ranged from 13 to 16, depending on season. California, Colorado, Connecticut, Georgia, Maryland, Michigan, Minnesota, New Mexico, New York, Ohio, Oregon, Tennessee, and Utah sites were included across the entire period.

† Surveillance typically occurs from October 1 through April 30 of each influenza season, but some seasons may have been extended beyond April 30 due to late seasonal influenza activity or for monitoring novel influenza strains (e.g., A(H5N1)). For this analysis, only admissions from October 1 through April 30 were included.

§ National Center for Health Statistics bridged-race population denominators (seasons before 2020) or U.S. Census Bureau unbridged race population denominators (2020–2025) were used for rate calculations.

¶ In 2021–22, all patients were sampled. In all other seasons from 2017–18 through 2024–25, 2%–100% of patients were randomly sampled for chart abstraction depending on season, site, age group, and outcome (died in hospital versus discharged alive); admission month also contributed to the sampling scheme during 2022–23 to 2024–25. In 2024–25, California, Colorado, Connecticut, Georgia, Maryland, Michigan, Minnesota, New Mexico, New York, Oregon, Tennessee, and Utah sampled patients for chart abstraction. In all seasons, age, sex, admission date, site, influenza testing results, and outcome were collected for all patients.

** 45 C.F.R. part 46.102(l)(2), 21 C.F.R. part 56; 42 U.S.C. Sect. 241(d); 5 U.S.C. Sect. 552a; 44 U.S.C. Sect. 3501 et seq.
